# Innovative Nasal Splitting Technique for Dual Rhinoplasty and Rhinophyma Management: A Case Report and Literature Review

**DOI:** 10.1055/a-2802-3112

**Published:** 2026-03-18

**Authors:** Michel L.H.T. Vaena, Bruno Benedetti Pinto, Carlos Weck Roxo

**Affiliations:** 1 Faculdade de Ciências Médicas – FCM/UERJ - Av. Prof. Manuel de Abreu, Rio de Janeiro State University - UERJ, Rio de Janeiro, Rio de Janeiro, Brazil; 2 Department of Plastic Surgery, Hospital Federal do Andaraí, Rio de Janeiro, Brazil; 3 Department of Plastic Surgery, Hospital Universitario Pedro Ernesto – UERJ, Rio de Janeiro, Brazil

**Keywords:** rhinophyma, rhinoplasty, nasal reconstruction, nose deformities, nose, cosmetic, others

## Abstract

Rhinophyma is a deforming disease that affects the nose mainly of Caucasian men. Progressive nasal skin thickening, telangiectasias, and hypertrophy of sebaceous appendages are frequently found in these patients. Many therapeutic modalities have been reported with good results. Among these, tangential excision is established as the surgical modality of choice. In advanced cases, it is not possible to distinguish nasal deformities resulting from rhinophyma from those resulting from malposition of the anatomic components that make up the nasal framework. We describe the use of a mixed technique in which the tangential excision and the open approach complement each other satisfactorily, restoring both shape and structure of the nose. This approach may represent a safe alternative for the simultaneous treatment of rhinophyma and a structured rhinoplasty, and further studies are needed for its full validation in clinical practice.

## Introduction

Rhinophyma is characterized by a progressive thickening of the nasal skin, especially on the lower half of the nose, affecting mostly Caucasian men between the fifth and seventh decades. Its pathophysiology is not fully understood, influenced by exogenous and endogenous factors, leading to chronic inflammation. 1 It may present with pronounced deformity and enlargement of the nasal lobule, causing the tip to fall and airway obstruction. 2 3 4 5 6 Different authors have proposed classifications for rhinophyma according to its severity. 7 8 9 El-Azhary et al have classified according to clinical presentation as minor (telangiectasias and mild thickening on the nose), moderate (thickening and early formation of lobules), or major degree (nasal hypertrophy and prominent lobules). 3 We describe a surgical approach for performing a structured rhinoplasty and simultaneously treating a major degree of rhinophyma.

## Case

**Fig. 1:**
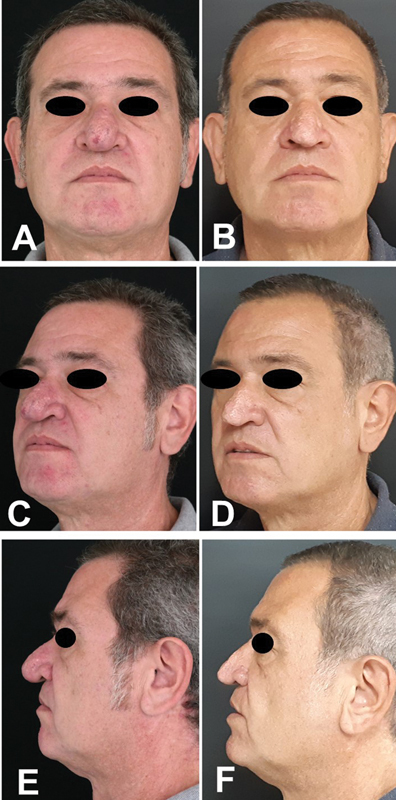
A 46-year-old man treated for rhinophyma. ( **A** ) Preoperative anterior view. ( **B** ) Postoperative anterior view after 1 year. ( **C** ) Preoperative left oblique view. ( **D** ) Postoperative left oblique view after 1 year. ( **E** ) Preoperative left lateral view. ( **F** ) Postoperative left lateral view after 1 year.

A 46-year-old male presented with nasal airway obstruction and aesthetic concerns. He was an active smoker and had undergone septoplasty 6 years prior. Although initial postoperative respiratory function improved, over the past 5 years, he experienced progressive enlargement of the nasal lobule, pendular movement of the nasal tip with head motion, and a gradual decline in nasal airflow. The patient had a marked rhinophyma that weighed on the nasal tip ( Fig. 1A, C, E ). On physical examination, lifting the nose tip (index finger to push up the nasal tip) caused evident respiratory improvement. Based on anamnesis and clinical examination, initially, a two-stage surgical treatment was proposed (first approaching the rhinophyma and then a rhinoplasty), but the patient refused and requested a single surgery. The patient provided written consent for the use of his image and medical records. We describe below our approach to rhinoplasty and rhinophyma in a single stage.

### Surgical Technique

**Fig. 2:**
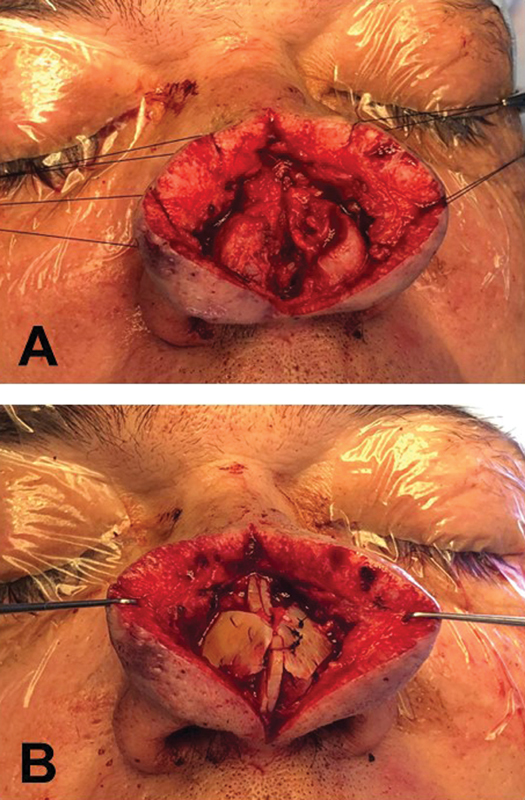
Intraoperative view prior to rhinophyma treatment. ( **A** ) The skin was incised longitudinally along the midline, from the dorsum to the columella, and the skin flaps were moved laterally to expose the entire cartilaginous structure of the nasal lobule. ( **B** ) Same view after the structured rhinoplasty, with costal cartilage grafts inserted and positioned. Note the thickness of the skin flaps before treating rhinophyma.

**Fig. 3:**
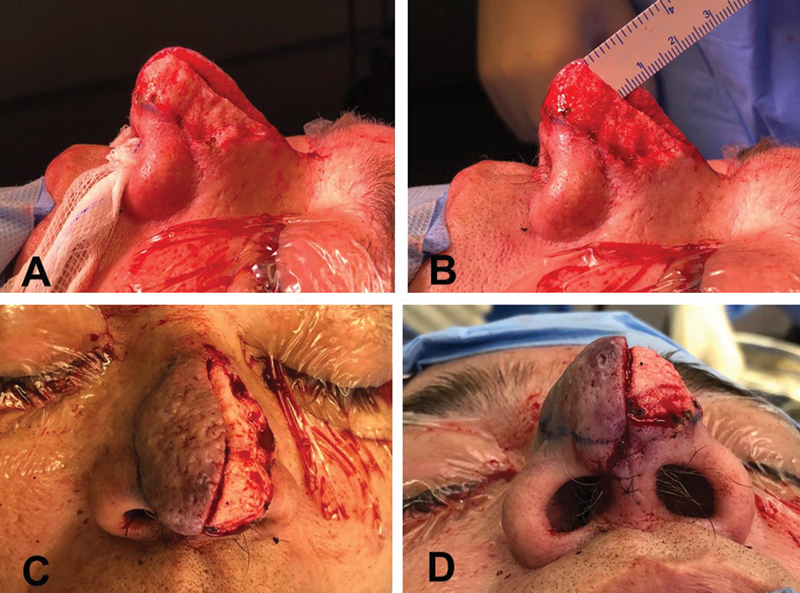
Intraoperative view during rhinophyma treatment. ( **A** ) After closure of the incision, the left skin flap was treated for rhinophyma and thinned to the desired level. ( **B** ) The main resecting parameter was the thickness of the contralateral skin flap. ( **C** ) Anterior view after thinning of the left skin flap. ( **D** ) Inferior view after thinning of the left skin flap.

With the patient under general anesthesia, the midline was marked from the radix to the columella. The nasal skin was infiltrated with epinephrine solution (1:80,000) using a 3-mL syringe with a hypodermic needle, distributing the solution evenly and symmetrically, avoiding distortions in the nasal shape. After vasoconstriction, the skin was incised longitudinally along the midline, from the dorsum to the columella, and the skin flaps were dissected and moved laterally to expose the entire cartilaginous structure of the nasal lobule. The exposure of the upper lateral cartilages, lower lateral cartilages, and distal septum allowed complete assessment of the nasal tip ( Fig. 2A ). At this point, it was determined that repositioning of the nasal anatomical structures was required. Given the insufficient septal cartilage due to the previous septoplasty, structural grafts were harvested from the right fifth rib ( Fig. 2B ). Spreader grafts, a septal extension graft, and alar batten grafts were fashioned and positioned with the objective of preserving external nasal valve patency and preventing nasal tip ptosis. Once the nose was restructured, the previous nasal splitting allowed direct visualization of the skin flaps in a transversal plane, with direct assessment of thickness, which was measured before closure of the incision, as a preliminary evaluation for the treatment of rhinophyma. The dermis of both skin flaps was sutured with separate stitches, using resorbable thread and inverted (buried) knots. After closure, first, the left flap was treated for rhinophyma with the scalpel, through successive removal of “slices,” thinning the skin flap to the desired level ( Fig. 3A ). At that moment, the main resecting parameter was the thickness of the contralateral skin flap, whose permanent visualization allowed to “sculpt” the proper shape of the nose without overdoing it, thus preserving the deeper pilosebaceous appendages and vascularization. This visual control of the flap thickness allowed satisfactory healing and minimized the risk of skin necrosis or exposure of the cartilage framework. Only after finishing the left skin flap, the right flap was treated the same way for symmetrization ( Fig. 3B–D ). Dermabrasion was used to complement the refinement of the skin. After treating the rhinophyma, non-adherent petrolatum-based dressings were applied, and no nasal packing was used postoperatively. Such dressings were used during the first 3 weeks postoperatively until complete re-epithelialization of the nose. The patient recovered uneventfully, with improvement in respiratory function and nasal aesthetics ( Fig. 1B, D, E ).

## Discussion

Rhinophyma is often related to end-stage acne rosacea, and clinical primary changes correlate with histopathological findings of sebaceous glands' hyperplasia, vascular dilatation, chronic inflammatory infiltrate, follicular cysts, elastosis, and moderate fibrosis in the dermis. 4 Pharmacological treatment of rhinophyma can be done topically (agents like metronidazole, ivermectin, azelaic acid) or systemically (medications such as isotretinoin, doxycycline, tamoxifen). 10 Non-surgical physical methods include radiotherapy (abandoned due to the risk of malignancy) and laser therapy excision. 3 5 6 7 11 12 There are several surgical methods described for the treatment of rhinophyma: Direct removal with the scalpel or disposable razor, electroscalpel, dermabrasion, and radiofrequency loops. 2 3 5 6 7 13 Other surgical methods using new debridement technologies have also been described. 14 15 Regardless of the technique used, all methods report excellent healing, each one presenting its advantages and disadvantages ( Table 1 ).

**Table 1:** Advantages and disadvantages of treatment methods for rhinophyma

Method	Principle/description	Advantages	Disadvantages	References
Cold-knife excision (scalpel)	Removal of hypertrophic tissue using a sharp blade; may include tangential excision and fine contouring	Low cost; short operative time; preserves pilosebaceous units; quicker re-epithelialization; good cosmetic contouring	Poor hemostasis; risk of intraoperative bleeding; possible unfavorable scarring; depth control can be imprecise; possible residual tissue/recurrence	1 4
CO _2_ laser ablation/vaporization	Laser vaporizes excess tissue; allows sculpting; hemostasis via coagulation	Precise decortication; controlled hemostasis; relatively bloodless surgical field; good in high-risk patients; efficient in contouring	More expensive; longer operative time; risk of thermal injury; postoperative hypopigmentation/erythema/scarring; no surgical specimen for histology in some cases	1 3
Er:YAG laser (dual-mode)	Erbium laser (short-/long-pulse) for tissue reduction with some hemostasis	Very good precision; reduced thermal damage compared to CO _2_ ; good intraoperative control; excellent aesthetic outcomes in mild-to-severe cases	Poorer hemostasis compared to CO _2_ ; possibly slower tissue removal; risk of hypopigmentation; more expensive; specialized training needed	4 6
Electrocautery/Electrosurgery/Radiofrequency (RF)	Use of electric current or heat to remove tissue/cauterize vessels; can include loop excision, etc.	Good hemostasis; shorter surgical time; cost-effective; good for contouring; can preserve deeper tissue; efficient removal; less expensive than laser methods; minimal bleeding when well done; outpatient possible	Risk of thermal damage; imprecise depth control; risk of cartilage necrosis; possible scarring; risk of over-removal leading to deformity/exposure; postoperative healing time/erythema; dependent on operator skill	7
Dermabrasion	Mechanical grinding/abrasion to smooth skin surface (often after debulking)	Smooth surface finish; good intraoperative control of contour; useful adjunct after tissue removal; relatively simple tools	Poor hemostasis; bleeding; risk of poor visibility during procedure; longer healing; potential for uneven removal; less used alone	2
Subunit method (flaps/staged reconstruction)	Surgical excision of tissue with reconstruction by nasal aesthetic subunits; may include flaps or grafts; addresses both tissue excess, skin redundancy, and structural support	Scars concealed along aesthetic subunit borders; restores framework; maximal tissue removal; improved nasal tip; better support; good for severe deformity; functional benefits (airway)	Technically demanding; more invasive; often two stages required; longer operative and healing times; higher risk of complications; requires surgeon experience; cost higher	20 21
Microdebrider-assisted excision	Use of microdebrider (rotating blade) for sculpting/contouring after bulk removal of tissue	Good contouring; preserves deeper layers; reduced intraoperative blood loss; shorter operative time; positive patient satisfaction; less damage to cartilage; useful especially for shaping tip and alae	Availability of equipment; operator learning curve; possibly more expensive; may still require medical resources/follow-ups; risk of residual tissue or needing revision	15

RF, radiofrequency; YAG, Yttrium Aluminum Garnet.

“High-risk patients” denote patients with comorbidities or bleeding risk.

Clinical outcomes (cosmetic satisfaction, functional airway) may vary by disease severity, surgeon skill, and postoperative care.

Statistical significance: In systematic reviews, cosmetic satisfaction and re-epithelialization time did not show large differences between excisional and laser techniques.

Rather than asking how to remove rhinophyma, one should focus on *how much* is to be removed. If the dermis has not been removed to its deeper layers, epithelialization should occur naturally from the dermal appendages, completely and without noticeable scars. So, if tangential excision is properly executed, excellent healing is the expected result, despite the method employed. 2 5 6 7 In a cost-effective approach, most surgeons do not request imaging tests to treat rhinophyma, with a high success rate and low complication rates. To our knowledge, no studies have investigated the use of imaging techniques, such as cutaneous ultrasound, for the preoperative assessment of rhinophyma. While clinical examination allows an initial estimation of tissue to be excised, the definitive extent is typically determined intraoperatively. Histopathological studies of non-rhinophymatous cadaveric specimens report nasal dermal thickness ranging from 1.0 to 1.8 mm across different subunits. Consequently, excessive flap thinning should be avoided, and a minimum dermal thickness of approximately 3 mm above the subcutaneous tissue is recommended. 16 This recommendation is supported by in vivo ultrasound measurements of nasal dermal thickness in living individuals. 17

Another important issue is that patients with long-standing rhinophyma may present secondary changes due to the weight of rhinophyma on the nasal lobule, which may distort the original position of the structural elements of the nasal tip, such as the alar cartilages and ligaments. It is not possible to distinguish these secondary alterations from the primary changes through clinical examination. Comparison with old photographs of the patient, prior to the development of rhinophyma, is a viable resource. Due to these changes, the collapse of the external nasal valve is frequent in severe cases of rhinophyma and may require internal structural intervention beyond just external debulking and thinning. Although in this case, description of the anamnesis and physical examination guided the therapeutic indications, ideally, nasal function should be evaluated pre- and postoperatively using objective methods, such as the Nasal Obstruction and Septoplasty Effectiveness Scale (NOSE) or 22-item Sino-Nasal Outcome Test (SNOT-22) 18 19 Whenever there is a functional indication for a rhinoplasty, for safety reasons, most surgeons choose to treat rhinophyma with sequential excision surgeries, and only to perform a rhinoplasty later, when nasal anatomy can be assessed more reliably. 2

For rhinoplasty purposes, our approach allows a complete exposure of the nasal lobule framework, and the centripetal vascularization of the skin flaps is minimally affected. To the best of our knowledge, this combined approach has not yet been described before. It also enables the precise removal of rhinophyma since the thickness of what is to be removed is previously measured. An open approach that allows nasal structuring with cartilage grafts has also been described by Hassanein et al. 20 21 This technique advocates debulking of the phymatous tissue from 2 or 3 mm below the epidermal surface down to the perichondrium, which may directly affect the vascular plexus, turning the flaps very susceptible to necrosis. In fact, in case of doubt regarding vascularization, the author recommends replacing the entire subunit with a graft. In our approach, the risk of removing the deeper layers of the dermis is minimized, thus avoiding situations such as skin necrosis, cartilage exposure, or unaesthetic scarring due to complicated secondary healing. Furthermore, certain changes found in the most severe cases of rhinophyma (such as pitting or cobblestoning) can only be approached by tangential excision from the external surface of the nose. For reasons described above, we believe the approach represents a safe alternative for the simultaneous treatment of both conditions, that is, rhinophyma and the falling of the nasal tip. Nonetheless, a solitary case report with a limited follow-up of 1 year precludes any definitive conclusions regarding the reproducibility and reliability of the technique, and further systematic investigations are warranted to substantiate its clinical validity.

We have described the use of a mixed technique in which the tangential excision and the open approach complement each other satisfactorily, restoring both shape and structure of the nose. After further studies, we believe this approach may be confirmed as a safe alternative for a structured rhinoplasty and simultaneous treatment of rhinophyma.
